# Ionic liquid-based multi-stage sugaring-out extraction of lactic acid from simulated broth and actual lignocellulosic fermentation broth

**DOI:** 10.1186/s40643-021-00481-4

**Published:** 2021-12-13

**Authors:** Xu Zhou, Yaqin Sun, Hongjun Zhan, Haijun Liu, Xiaoyan Wang, Yang Xu, Yi Li, Zhilong Xiu, Yi Tong

**Affiliations:** 1 School of Bioengineering, Dalian University of Technology, No.2 Linggong Road, Ganjingzi District, Dalian, Liaoning 116024 People’s Republic of China; 2 Jilin COFCO Biochemistry Co., Ltd. (National Engineering Research Center of Corn Deep Processing), Changchun, Jilin 130033 People’s Republic of China

**Keywords:** Ionic liquid, Sugaring-out extraction, Lactic acid, Multi-stage, Fermentation broth

## Abstract

In this study, ionic liquid-based sugaring-out extraction was developed to separate lactic acid from the synthetic solution and actual lignocellulosic fermentation broth. Except for [E_OH_mim]BF_4_, the ILs with BF_4_^−^ and OTF^−^ anion can form aqueous two-phase system (ATPS) with the aid of saccharides. With the same kind of saccharides, the ATPS formation ability of ILs could be promoted by increasing the side-chain length of ILs in the order of [Hmim]BF_4_ ≈ [Bmim]BF_4_ ˃ [Emim]BF_4_ due to the decrease in ILs’ kosmotropicity. On the other hand, for the same type of ILs, an ATPS was formed more easily with glucose than with xylose. When IL concentration varied from 35% (w/w) to 40% (w/w) at a low glucose concentration of 15% (w/w), an interesting phase reversal was observed. When lactic acid was undissociated at pH 2.0, 51.8% LA and 92.3% [Bmim]BF_4_ were partitioned to the top phase, and 97.0% glucose to the bottom phase using an ATPS consisting of 25% (w/w) glucose and 45% (w/w) IL. The total recovery of LA would increase to 89.0% in three-stage sugaring-out extraction from synthetic solution. In three-stage sugaring-out extraction from the filtered and unfiltered fermentation broth obtained via simultaneous saccharification and co-fermentation (SSCF) of acid-pretreated corn stover by the microbial consortium, the total recovery of LA was 89.5% and 89.8%, respectively. Furthermore, the total removal ratio of cells and pigments from the unfiltered broth was 68.4% and 65.4%, respectively. The results support IL-based sugaring-out extraction as a potential method for the recovery of lactic acid from actual fermentation broth.

**Graphical Abstract:**

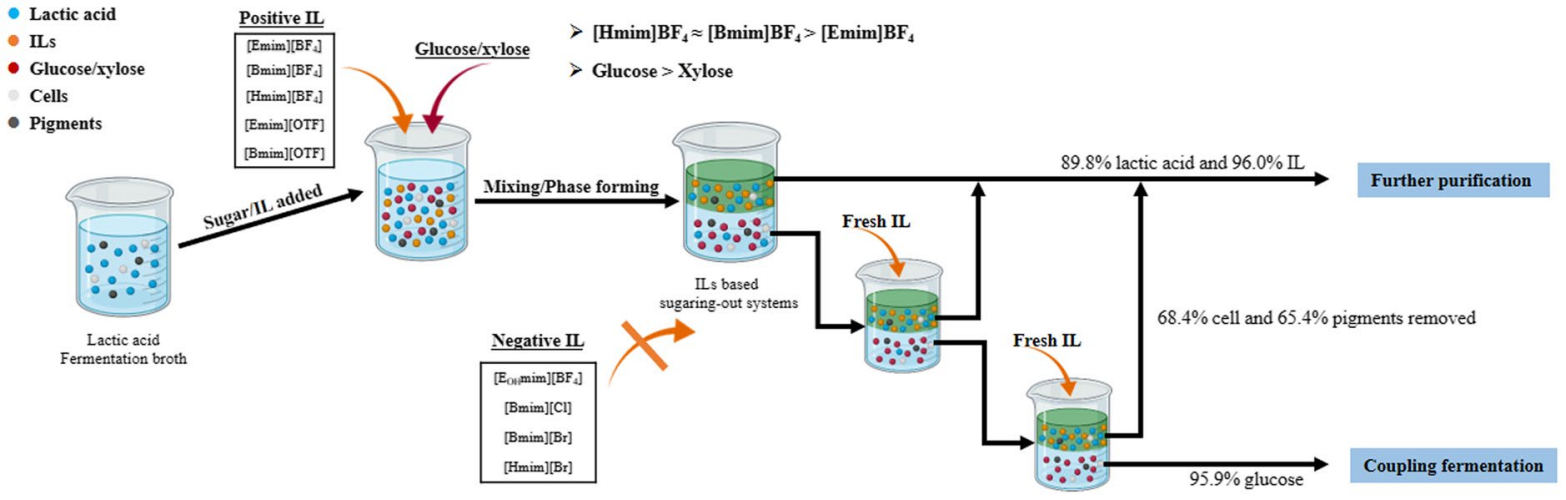

## Highlights

- IL-based sugaring-out extraction was applied to separate LA from fermentation broth
- Different ILs and saccharides consisting systems were examined
- The ability to form ATPS is in the order of [Hmim]BF4 ≈ [Bmim]BF4˃[Emim]BF4
- The partition behaviors of LA, [Bmim]BF4, and saccharides were evaluated
- LA recovery of 89.8% was obtained with removal of 68.4% cells and 65.4% pigments

## Introduction

Lactic acid (LA), an important platform chemical, is widely used in the food, pharmaceutical, and chemical industries, particularly as a monomer of biodegradable and biocompatible polylactic acid (Ahmad et al. 2020). Low-cost lignocellulosic feedstocks have received a lot of attention as an alternative carbon source for LA production because they are readily available, sustainable, and renewable (Isikgor and Becer 2015; Yuan et al. 2018). However, lignocellulose-derived lactic acid may produce more impurity components in broth than starch or sugar-based lactic acid production, such as lignin, protein, other organic acid, furan derivatives, and pigments. More complex processes and higher costs are required to separate LA from lignocellulose-derived broth (Xu et al. 2018). Developing an efficient and cost-effective method for separating second-generation LA is both necessary and challenging. As a result, economical and efficient recovery and purification processes for lignocellulosic lactic acid are desired.

Various separation and purification technologies of LA, including precipitation (Meng et al. 2020), extraction (Matsumoto et al. 2003; Oliveira et al. 2012), distillation (Komesu et al. 2017), electrodialysis (Choi et al. 2002), aqueous two-phase (Lan et al. 2019; Xu et al. 2018; Yan et al. 2016), adsorption (Tang et al. 2013; Zhang et al. 2018), and liquid membranes (Matsumoto et al. 2020), have all been thoroughly studied and demonstrated. Among these methods, aqueous two-phase (ATP) is recognized as a promising method for separating biochemicals because of their high selectivity, easy scale-up, and continuous operation mode (Claudio et al. 2014). Up to now, the majority of lactic acid purification ATP systems (ATPSs) were based on polyethylene glycol (PEG)/salt systems (Yankov et al. 2008). Because of the high cost of the polymers and the difficulty in isolating the extracted molecules from the polymer phase by back-extraction, these systems are unlikely to be used to produce bulk chemicals on a large scale (Aydogan et al. 2011). Recently, salting-out extraction systems, comprising short-chain alcohol and inorganic salts, have been successfully used to separate lactic acid due to their high separation efficiency, low cost, low interfacial tension, and ease of scale-up (Aydogan et al. 2011; Fu et al. 2015; Song et al. 2013; Yan et al. 2018, 2016). Salting-out extraction could recover over 87% lactic acid using the system of 18.40% (w/w) dipotassium hydrogen phosphate and 30.23% (w/w) ethanol (Aydogan et al. 2011). Salting-out extraction ATPS usually happens at high salt concentrations, which may induce unwanted chemical reactions and may not be good for the separation of labile biological components (Lightfoot and Moscariello 2004; Wang et al. 2008a). The recovery and reuse of inorganic salt become the critical factor for the application of salting-out extraction. To avoid using a large amount of salts and to reduce wastewater generation, sugars as the substrates in fermentations for sugar-based chemicals can replace salts to trigger two-phase separation. Sugaring-out was firstly proposed by adding a mono-sugar or a disaccharide into an acetonitrile–water mixture (Wang et al. 2008a, b). Compared to sugaring-out system, the sugaring-out extraction ATPS consisting of sugars and solvent has potential advantages in hydrophilic compounds separation (Dai et al. 2015, 2017; Sun et al. 2019; Yan et al. 2016). A sugaring-out extraction system consisting of isopropanol and glucose was investigated to separate lactic acid (Yan et al. 2016). LA recovery of 84.27% with a partition coefficient of 1.39 was obtained by a system consisting of 12% (w/w) glucose and 40% (w/w) isopropanol. Lactic acid extraction yields were comparable for short-chain alcohol-based salting-out extraction and sugaring-out extraction ATPS. Organic solvents were generally selected as the extractants among the mentioned salting-out extraction and sugaring-out extraction. The high toxicity and volatility of organic solvent limit their use on an industrial scale. In order to minimize environmental impacts about the use of volatile organic compounds, ionic liquid (IL), a green solvent with low volatility at ambient conditions, low toxicity, biocompatibility, and biodegradability, has been used in separation processes (Han and Armstrong 2007). IL could offer an alternative to organic solvents to develop a suitable extraction process because the chemical and physical properties of ILs are tunable by choosing the appropriate anion and cation pair (Muller et al. 2013). Therefore, IL has emerged as a valuable option for forming an ATPS because they typically provide faster phase separation, a significant reduction in viscosity, and the ability to tailor the coexisting phases’ polarities in such a way that complete extraction efficiencies can always be predicted (Claudio et al. 2014; Dai et al. 2021). Because of these characteristics, as well as the vast possibility of their ions rearrangement, ILs have an outstanding ability to extract and purify biochemicals. Ionic liquid-based ATPS was demonstrated and applied to separate biochemicals, e.g., succinic acid (Pratiwi et al. 2015; Sun et al. 2018) and 1,3-propanediol (Muller et al. 2013). However, the ionic liquid-based sugaring-out extraction system was never reported to separate lactic acid from actual lignocellulosic fermentation broth.

Hence, the current research sought to investigate the ability of water-miscible ionic liquids and sugars to build aqueous two-phase systems for lactic acid separation. First, the ability of ionic liquids composed of different anions and cations to form an ATPS with the aid of saccharides was investigated. Furthermore, the main factors influencing extraction efficiency were investigated, including IL concentration, saccharides type and concentration, and the dissociation of lactic acid or not. Final, the multi-stage extraction system was applied to extract lactic acid from the corn stover-derived fermentation broth. The partition behaviors of the cell, proteins, and pigments were also studied.

## Materials and method

### Chemicals

The investigated ILs are listed, together with their abbreviations and chemical structures, in Table 1. All of the ILs were purchased from Chengjie Chemical Co., Ltd. (Shanghai, China) with a purity of greater than 99%. Glucose, xylose, lactic acid, and other chemicals were purchased from Sinopharm Chemical Reagent Co., Ltd (Shanghai, China) and were analytical grade.

**Table 1:** Abbreviations and chemical structures of the investigated ILs

Abbreviation	IL	Chemical structure	Forming ATPS
[Emim]BF_4_	1-ethyl-3-methylimidazolium tetrafluoroborate		+
[Bmim]BF_4_	1-butyl-3-methylimidazolium tetrafluoroborate		+
[Hmim]BF_4_	1-hexyl-3-methylimidazolium tetrafluoroborate		+
[E_OH_mim]BF_4_	1-thoxy-3-methylimidazolium tetrafluoroborate		−
[Emim]OTF	1-ethyl-3-methylimidazolium trifluoromethanesulfonate		+
[Bmim]OTF	1-butyl-3-methylimidazolium trifluoromethanesulfonate		+
[Bmim]Cl	1-butyl-3-methylimidazolium chloride		−
[Bmim]Br	1-butyl-3-methylimidazolium bromide		−
[Hmim]Br	1-hexyl-3-methylimidazolium bromide		−

+ represents that it can form an aqueous two-phase, and −represents that it cannot form an aqueous two-phase

### Simulated broth and lignocellulosic fermentation broth

Simulated broth, including 89.25 g/L lactic acid, was prepared. The fermentation broth was obtained from simultaneous saccharification and co-fermentation (SSCF) of dilute acid-pretreated corn stover by microbial consortium (Sun et al. 2021). Corn stover was milled into particles with size < 2 mm and then was pretreated by 1% (v/v) dilute H_2_SO_4_ solution with 10% (w/v) of dry biomass loading. The SSCF process was operated for 110 h, and the final concentrations of LA and residual xylose in the fermentation broth were 34.32 g/L, and 17.76 g/L, respectively. The fermentation broth was centrifuged for 20 min at 4500 rpm, which is defined as unfiltered broth. Then the unfiltered broth was filtered by a cellulose triacetate hollow fiber dialyzer (Sureflux-150G) which is defined as filtered broth. As a result, the final concentration of LA and residual xylose in the filtered broth was 29.80 and 15.50 g/L, respectively.

### Binodal curves

In this study, the binodal curves of ILs with saccharides at 293.15 K were obtained by the cloud point method according to the published study (Fu et al. 2015). Glucose or xylose solution with varying concentrations was titrated dropwise with ILs until the solution became turbid. The compositions of these mixtures were noted and determined by an analytical balance.

### IL-based sugaring-out extraction experiments

Simulated fermentation broth as described in “Simulated broth and lignocellulosic fermentation broth” Section was used to investigate the influences of sugaring-out extraction system for the partition behaviors of LA, IL, and saccharides in one-stage extraction. The glucose or xylose was dissolved in the simulated and actual fermentation broth to obtain a saccharide mixture. Following that, [Bmim]BF_4_ was added into the saccharide mixture and mixed thoroughly by a vortex mixer. The obtained mixture was allowed to stand for 8 h.

The optimized sugaring-out extraction system was then developed in three-stage for simulated broth, unfiltered fermentation broth, and filtered fermentation broth. For the first-stage extraction, 45% (w/w) IL and 25% (w/w) glucose were added to the broth at pH 2.0. The glucose-rich phase was then prepared for the second and third stages of extraction. Only fresh IL was added in the second and third stages to achieve a sugaring-out extraction system that contained 45% (w/w) IL.

The phase ratio (*R*), partition coefficient (*K*), and extractability (*Y*) for each stage of sugaring-out extraction were defined as our previous report (Sun et al. 2018).

The removal ratio of cells, soluble proteins, and pigments (${D}_{Mi}$) for each stage was defined as follows:

$$~D_{{Mi}} = \left( {1 - {\raise0.7ex\hbox{${M_{{Ti}} }$} \!\mathord{\left/ {\vphantom {{M_{{Ti}} } {M_{{Wi}} }}}\right.\kern-\nulldelimiterspace} \!\lower0.7ex\hbox{${M_{{Wi}} }$}}} \right) \times 100\% ~~~i = 1,2,3$$

where ${M}_{Ti}$ is the mass of cells, proteins, and pigments in the top phase and ${M}_{Wi}$ is total mass of cells, proteins, and pigments in the added solution for each stage of sugaring-out extraction system, respectively.

The total extractability of lactic acid and glucose for the three stages of sugaring-out extraction could be calculated as follows:

$$TY=\left\{{Y}_{1}+{Y}_{2}\left(1-{Y}_{1}\right)+{Y}_{3}[1-{Y}_{1}-{Y}_{2}\left(1-{Y}_{1}\right)]\right\}\times 100\%,$$

where ${Y}_{1}, { Y}_{2},{{\rm and}\,Y}_{3}$ represent the extraction yield of the 1st, 2nd, and 3rd stages, respectively. The average extractability of three stages was used to calculate the total extractability of IL.

The total removal ratio of cells, soluble proteins, and pigments was defined as follows:

$${TD}_{M}=\prod_{i=1}^{3}{D}_{Mi}.$$

### Analytical methods

The concentrations of lactic acid, glucose, xylose, and ILs were analyzed using HPLC equipped with an Aminex HPX-87H column with a column temperature of 65 °C. Sulfuric acid (5 mmol/L) was the mobile phase with a flow rate of 0.6 mL/min. The biomass concentration was measured by absorbance at 600 nm using a spectrophotometer. The concentration of soluble proteins was determined by the BCA Protein Colorimetric Assay Kit. The pigments were analyzed and measured by UV spectrophotometry at 320 nm. Each experiment was carried out in triplicate and the mean experimental values are given in the tables and figures.

## Results and discussion

### ATPS formation ability of ionic liquids/ saccharides

To effectively apply IL-based sugaring-out extraction systems as extractive platforms, their phase diagrams must be established experimentally. Table 1 shows the results of ILs forming aqueous two-phase systems with saccharides. The ILs with BF_4_^−^ anion, with the exception of [E_OH_mim]BF_4_ and OTF^−^ anion, can form ATP systems with glucose or xylose. However, the investigated ILs with Br^−^ and Cl^−^ anion cannot form an ATPS with saccharides. Previous research found that 1-alkyl-3-methyl imidazolium chloride ([C_*n*_mim]Cl, *n* = 2 to 10) and 1-alkyl-3-methyl imidazolium bromide ([C_*n*_mim]Br, *n* = 2 to 10) never form ATPS with carbohydrate in temperatures ranging from 242.15 K to 373.15 K, whereas [C_*n*_mim]Cl (n = 1) and [C_*n*_mim]Br (n = 1) can form ATPS with sucrose (Chen et al. 2009; Wu et al. 2008a). BF_4_^−^ is generally recognized as a chaotropic anion, and the imidazolium ring is recognized as a kosmotropic cation. However, the effect of imidazolium-based cation hydration is inconclusive when compared to the hydration of BF_4_^−^ (Wu et al. 2008b). As a result, it appears reasonable to consider imidazolium tetrafluoroborate as a chaotropic IL. Among the four imidazolium tetrafluoroborate ILs studied, [E_OH_mim]^+^ is more kosmotropic than [Emim]^+^. Therefore, it is difficult to form an ATPS for [E_OH_mim]^+^ with saccharides. Moreover, the anion Cl^−^ and Br^−^ are more hydrophilic than BF4^−^, it is also difficult to form ATPS for Cl^−^ and Br^−^ anion when mixed with kosmotropic solutions.

**Fig. 1:**
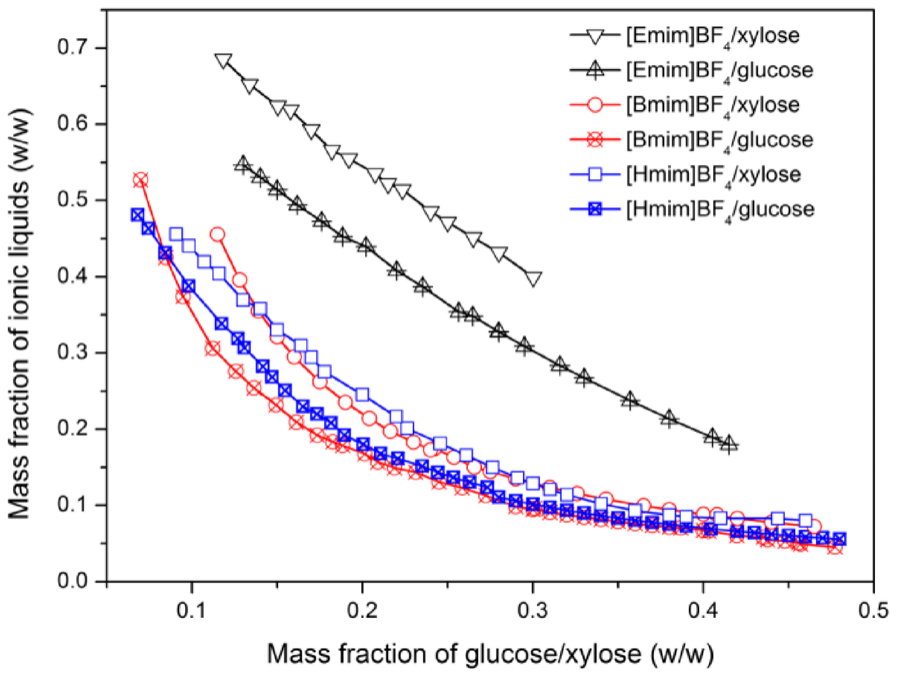
Binodal curves for BF_4_^−^ anion ILs + glucose + H_2_O system, BF_4_^−^ anion ILs + xylose + H_2_O system at 298.15 K

Figure 1 depicts the binodal curves of the BF_4_^−^ anion ATPS with glucose or xylose. When the same types of saccharides investigated, it was discovered that increasing the side-chain length of ILs could promote the formation of an ATPS. The kosmotropicity of ILs decreased as their side-chain length increased. Therefore, the phase separation abilities of ILs with the same kind of saccharides are in the order of [Hmim]BF_4_ ≈ [Bmim]BF_4_ ˃ [Emim]BF_4_ as shown in Fig. 1. Furthermore, -OH groups on sugar molecules that destroy the natural hydrogen-bonded water network, as well as the stereochemistry of saccharides, have a significant impact on the formation of ATPS. The more hydroxyl groups there are in a saccharide, the more kosmotropic the saccharide. The mean number of the equatorial hydroxyl group of glucose and xylose are 4.56 and 3.67, respectively (Wu et al. 2008b). Therefore, glucose is more kosmotropic than xylose, and the ability to form an ATPS of glucose is stronger than that of xylose. Previous reports systematically studied the liquid–liquid equilibria of the [Bmim]BF_4_ with fructose and sucrose system (Wu et al. 2008a, b; Zhang et al. 2007). It was discovered that [Bmim]BF_4/_fructose and [Bmim]BF_4/_sucrose ATPS can be formed over a wide component range, with the ability to form ATPS in the order of sucrose > glucose > xylose > fructose.

For the production of LA from lignocellulosic biomass, a mixture of glucose and xylose is obtained, as we all know. Due to the strong ability of [Bmim]BF_4_ to form ATPS with glucose and xylose, the partition behaviors of LA, saccharides, and [Bmim]BF_4_ were investigated and analyzed in the following study.

### Partition behavior of LA, saccharides, and IL in ATPS of [Bmim]BF_4_/saccharide

**Fig. 2:**
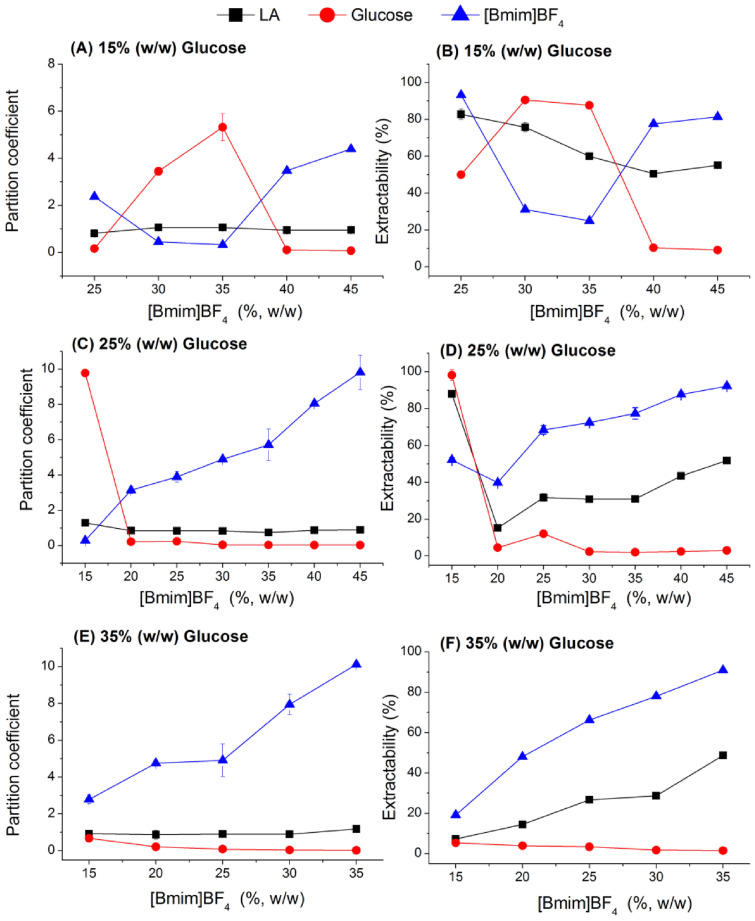
The effect of glucose and [Bmim]BF_4_ concentration on the partition behaviors of LA, glucose, and IL in [Bmim]BF_4_/glucose system at pH 2.0

**Fig. 3:**
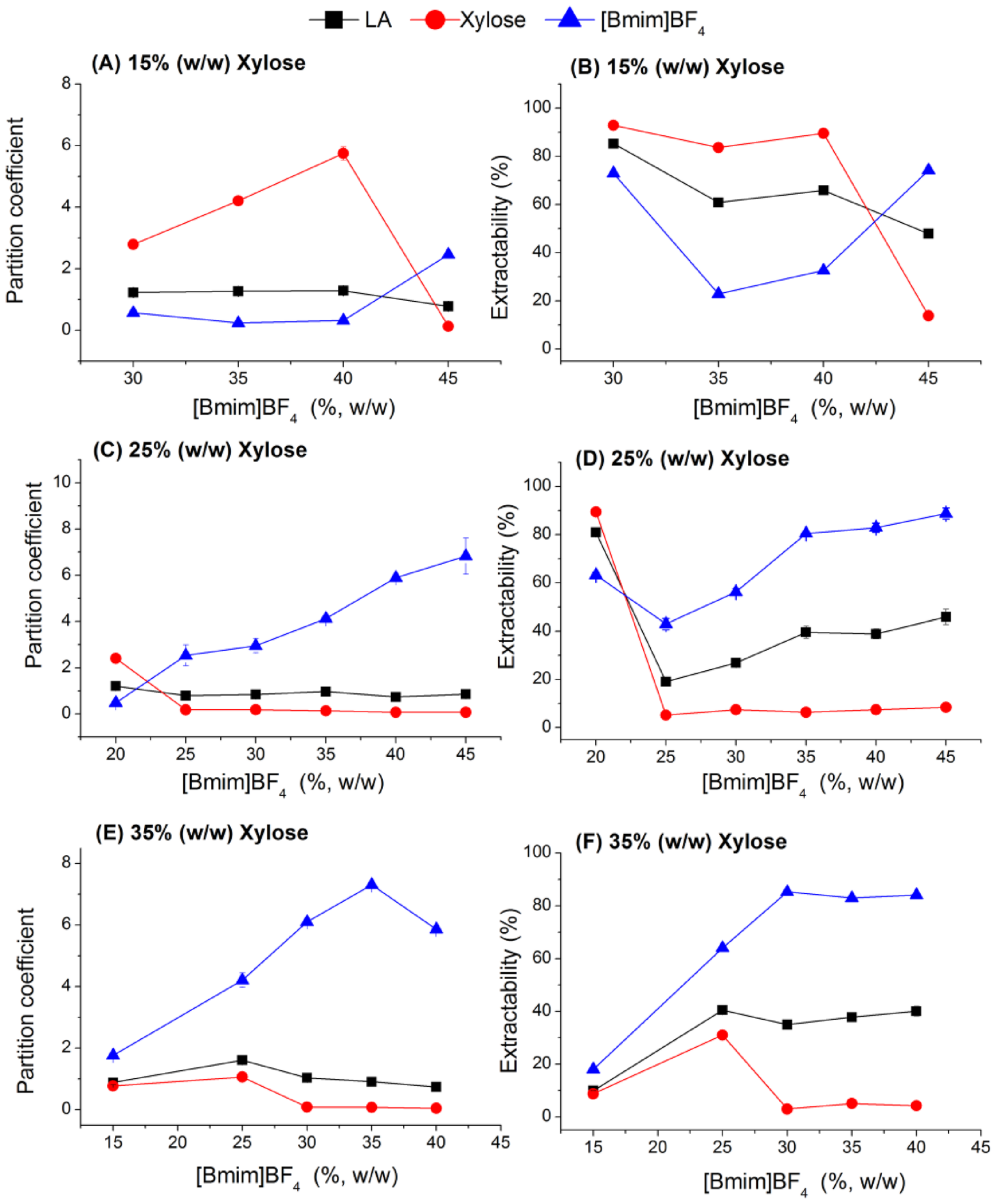
The effect of xylose and [Bmim]BF_4_ concentration on the partition behaviors of LA, xylose, and IL in [Bmim]BF_4_/xylose system at pH 2.0

In this study, the aqueous two-phase systems of [Bmim]BF_4_/glucose and [Bmim]BF_4_/xylose were investigated to extract lactic acid from the simulated broth. The effect of [Bmim]BF_4_ and saccharides concentration on the distribution behaviors of LA, [Bmim]BF_4_, and saccharides was investigated and is shown in Figs. 2 and 3. The IL was apt to the top phase, while glucose and xylose were apt to the bottom phase, especially in the high mass fraction of saccharide and IL in ATPS. An apparent phase reversal was observed when the IL concentration was varied from 35% (w/w) to 40% (w/w) at a low glucose concentration of 15% (w/w). The concentration of ionic liquid required for phase reversal gradually decreased as the concentration of glucose increased. When the ATPS contained 25% (w/w) glucose and 15% (w/w) [Bmim]BF_4_, over 98% glucose and 52% IL were extracted to the top phase. When the IL mass fraction was increased to 20% (w/w), only 5% glucose was distributed to the top phase. In the xylose-based sugaring-out extraction system, phase reversal was also observed (Fig. 3D). A high phase ratio was achieved while abundant glucose and IL were extracted to the top phase, resulting in high lactic acid extractability. The residual glucose or xylose was concentrated on the top phase, which hampered further purification of LA.

At a mass fraction of saccharide greater than 25% (w/w) and IL greater than 20% (w/w), the partition coefficient of LA and [Bmim]BF_4_ increased with increasing IL concentration. The increase in the partition coefficient for [Bmim]BF_4_ was obviously accompanied by a small increase in the partition coefficient for LA. At the same conditions, the partition coefficient of saccharide exhibited the opposite trend. When the IL concentration was increased from 20% (w/w) to 45% (w/w) at a 25% (w/w) glucose concentration, the phase ratio increased from 0.21 to 1.22. As a result, at a sugaring-out extraction system consisting of 25% (w/w) glucose and 45% (w/w) IL, approximately 51.8% LA and 92.3% [Bmim]BF_4_ were extracted to the top phase and 97.0% glucose was extracted to the bottom phase. The extraction efficiency of the xylose-based ATPS is lower than that of the glucose-based ATPS. About 45.9% LA and 88.7% [Bmim]BF_4_ were extracted to the top phase, and 91.6% xylose was extracted to the bottom phase at a sugaring-out extraction system consisting of 25% (w/w) xylose and 45% (w/w) IL.

The phase ratio decreased as the concentration of saccharides increased, and the partition coefficient of [Bmim]BF_4_ increased rapidly. As a result, the extractability of IL increased from 25.0% to 91.0% when the glucose concentration was increased from 15% (w/w) to 35% (w/w) with [Bmim]BF_4_ at 35% (w/w). The partition coefficient of LA showed a nearly constant trend, and the extractability decreased from 60.1% to 48.8% due to a decrease in phase ratio from 1.41 to 0.81. Glucose was apt to the top phase at a low mass fraction of glucose in the sugaring-out extraction system. As a result, a high partition coefficient of glucose was obtained in the low mass fraction of glucose and IL selected. The partition coefficient for glucose reached up to 5.32 at 15% (w/w) glucose and 35% (w/w) [Bmim]BF_4_ selected. An increase in the glucose concentration caused as decrease in the partition coefficient of glucose to 0.04 at 25% (w/w) glucose and 35% (w/w) [Bmim]BF_4_ selected. Accordingly, 87.7% and 2.0% glucose was extracted to the top phase in 15% (w/w) glucose and 25% (w/w) glucose ATPS, respectively.

In a previous study, a sugaring-out system consisting of 45% (w/w) [Bmim]BF_4_ and 30% (w/w) glucose at pH 2.0 yielded a relatively high partition coefficient of 2.53 and an extractability of 75.96% for succinic acid (Sun et al. 2018). Lactic acid had a lower extractability than succinic acid. Lactic acid's kosmotropic ability was more potent than succinic acid's due to the greater number of hydroxyl groups in lactic acid. As a result, lactic acid was more easily distributed to the kosmotropic phase than succinic acid because [Bmim]BF_4_ was chaotropic and saccharide was kosmotropic.

**Fig. 4:**
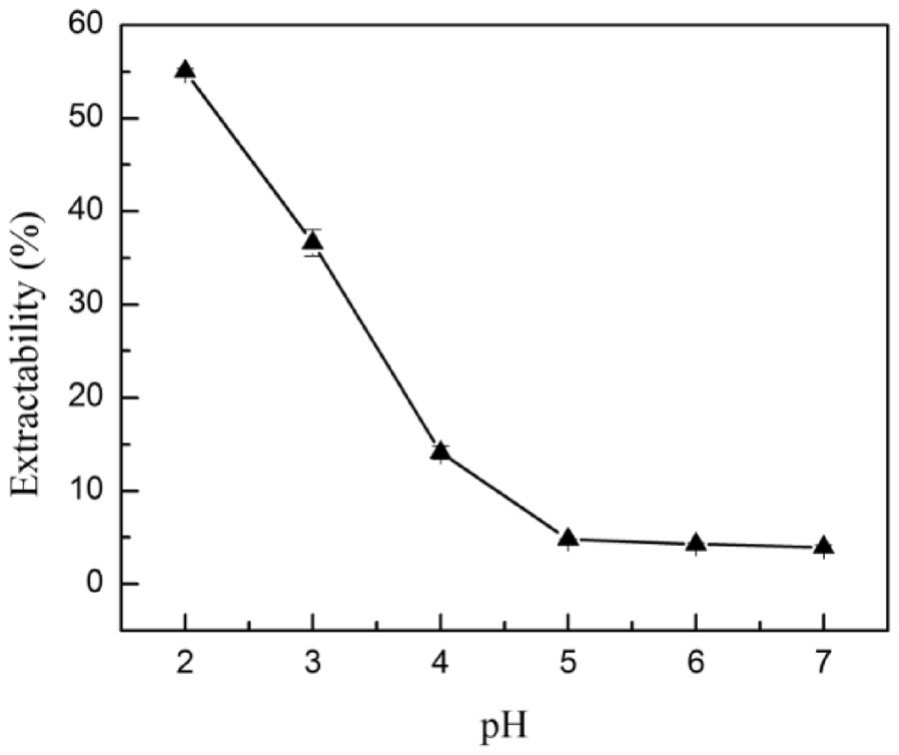
Relationship between equilibrium pH and extractability of lactic acid in 45% (w/w) [Bmim]BF_4_ and 25%(w/w) glucose system

Based on the partitioning behaviors of LA, saccharide, and [Bmim]BF_4_ in sugaring-out extraction systems, a 25% (w/w) glucose and 45% (w/w) [Bmim]BF_4_ system was chosen for further investigation. Figure 4 depicts the relationship between the equilibrium pH and the extractability of LA. Our previous study found that a pH lower than the pK of organic acid causes high extractability and is beneficial for organic acid recovery in sugaring-out and salting-out extraction systems. The effect of pH on LA partition behavior in ionic liquid-based sugaring-out extraction systems was similar. Lactic acid has a dissociation constant of about 3.86. As a result, 55.1% LA was recovered to the IL-rich phase at pH 1.94, whereas only 14.1% LA was extracted to the IL-rich phase at pH 3.96. Even when the pH was raised to 6.94, only 4.0% of the LA was distributed to the top phase. The pH change had almost no effect on the partition behavior of glucose and [Bmim]BF_4_. About 98% [Bmim]BF_4_ was distributed to the top phase, and 90% glucose was distributed to the bottom phase when the pH was varied from 1.94 to 6.94.

The mechanism of ATPS formation and the partition behavior of lactic acid at different pHs can be explained by the hydration theory. As a process of ionic hydration, when a saccharide is dissolved in an aqueous solution, its ions are surrounded by a layer of water molecules. The formation of IL and saccharide ATPS may be considered to be a competition between the hydrophilic IL and the saccharide for the water molecules. The competition was won by the ions which have a stronger affinity for the water. As illustrated in “ATPS formation ability of ionic liquids/ saccharides” Section, ILs tend to be chaotropic (water-destructuring) salts and saccharides tend to be kosmotropicity (water-structuring). Therefore, water molecules migrated away from the ions of the IL to those of the saccharide. As a result, the hydration and the solubility of the ILs decreased. Consequently, an IL-rich phase separates from the rest of the solution of saccharide. The hydrophobicity parameters (LogP) of lactic acid and sodium lactate were − 0.548 and − 1.883, respectively. The large the LogP, the more hydrophobic it is. Therefore, lactic acid was more hydrophobic than sodium lactate. As a result, lactic acid was apt to the chaotropic IL-rich phase and lactate was apt to the kosmotropic saccharide-rich phase. It is also stated that in the alcohol/IL-rich phase, undissociated organic acid may be integrated into the hydrogen-bonding network (Sun et al. 2018; Wakisaka and Ohki 2005).

### Multi-stage sugaring-out extraction of LA from synthetic solution and fermentation broth

In this study, multi-stage separation of lactic acid from simulated broth and actual fermentation broth obtained via SSCF process from corn stover by microbial consortium was investigated and applied. The outcomes were compared and are shown in Table 2. Through three stages of sugaring-out extraction of filtered and unfiltered fermentation broth, the total LA extractability of 89.5% and 89.8% was obtained, respectively, which is comparable to the total LA extractability of 89.0% from the simulated fermentation broth. It was worth noting that the residual xylose in the fermentation broth, which accounted for about 0.53 percent (w/w), aided the extraction efficiency of LA. Furthermore, the salts in the fermentation medium improved the extractability of lactic acid.

**Table 2:** Multi-stage extraction lactic acid by [Bmim]BF4/glucose systems from synthetic solution and lignocellulosic biomass-derived broth at pH 2.0

Solution Stage		R	K			Y (%)			D_M_ (%)	
			LA	Glucose	IL	LA	Glucose	IL	Cell	Pigments
Synthetic solution	1st	1.16	0.99	0.02	10.02	53.4	2.7	92.1	–	–
	2nd	1.11	0.99	0.02	11.40	52.2	2.5	92.7	–	–
	3rd	1.04	1.00	0.02	12.27	50.8	1.8	92.7	–	–
	Total	–	–	–	–	89.0	6.8	92.5	–	–
Filtered broth	1st	1.12	0.87	0.02	12.40	49.2	1.8	93.3	84.9	72.8
	2nd	1.05	1.06	0.02	23.86	52.8	2.0	95.1	90.9	86.5
	3rd	1.08	1.18	0.03	41.15	56.2	3.1	97.4	91.5	90.5
	Total	–	–	–	–	89.5	6.6	95.2	70.6	57.0
Unfiltered broth	1st	1.03	0.94	0.02	12.62	49.2	1.7	92.8	83.9	78.5
	2nd	1.02	1.10	0.01	33.99	52.1	1.1	97.1	88.8	90.0
	3rd	0.95	1.51	0.02	54.04	58.2	1.4	98.0	91.9	92.7
	Total	–	–	–	–	89.8	4.1	96.0	68.4	65.4

The synthetic solution and fermentation broth contain 89.25 g/L and 34.32 g/L lactic acid, respectively. 45% (w/w) [Bmim]BF_4_ and 25% (w/w) glucose were used

As a result, the total extraction yield of LA for the actual fermentation broth was comparable to that of the simulated broth, which only contained lactic acid. In a previous report, inorganic salts were considered to assist the extraction to enhance extractability of succinic acid in a solvent-based sugaring-out extraction system. When the mass fraction of (NH_4_)_2_SO_4_ was increased from 1 to 9% (w/w), the recovery of succinic acid from the broth increased from 87.6% to 89.6%. The addition of (NH_4_)_2_SO_4_ improves the recovery of succinic acid and enhances the *t*-butanol distributed to the top phase and glucose distributed to the bottom phase. In this study, salts in the lignocellulose-derived fermentation broth had a non-negligible effect on the partition behavior of [Bmim]BF_4_. The ionic liquid with BF_4_^−^ anion showed the ability to form two-phase not only with glucose but also with salts (Sun et al. 2018; Li et al. 2009). The mechanism of ATPS formation is both under the label of hydration theory (Trindade et al. 2007). The kosmotropic ions of salts, PO_4_^3−^, CO_3_^2−^, SO_4_^2−^, H_2_PO_4_^−^, OH^−^, and Cl^−^, which exhibit stronger interaction with water molecules, are beneficial to the ATPS formation. As the [Bmim]BF_4_ is a chaotropic (water-destructuring) IL, it was apt to the top phase with the aid of salts in the fermentation medium. As a result, more than 95% [Bmim]BF_4_ was extracted to the top phase in unfiltered and filtered fermentation broth during the second and third stages, which was higher than in simulated broth. The use of [Bmim]BF_4_ as phase-forming components in sugaring-out extraction systems has led to outstanding extraction performances for LA. Nevertheless, IL regeneration, recycling, or reuse lagged behind and still remain a challenging task toward the development of greener cost-effective processes. In previous studies, electrodialysis, back-extraction using Na_2_CO_3_, nanofiltration membrane, ion-exchange resin, and rotary evaporation stripped with compressed air were developed to separate and recover the targets and ILs (Claudio et al. 2014; Muller et al. 2013; Sui et al. 2018; Wang et al. 2012, 2016). Over 94% IL was recovered via back-extraction (Claudio et al. 2014). More than 95% of [Bmim]BF_4_ could be absorbed by the resins of Amberlite IR 120Na at 20 °C with contact time of 30 min (Sui et al. 2018). Moreover, the recovered IL was as effective as fresh IL for 1,3-propanediol extraction (Muller et al. 2013). In this study, these strategies for LA and IL recovery might be reasonable and optional.

Furthermore, approximately 95% glucose was present in the bottom phase at the same operation condition, which can be reutilized as a fermentation medium to achieve fermentation and separation coupling. In our previous work, the glucose-rich bottom phase in organic solvent-based sugaring-out extraction systems has been successfully reutilized for lactic acid, 2,3-butandiol, and acetoin fermentation (Dai et al. 2015, 2017; Yan et al. 2016). The bacterial growth, glucose consumption, and lactic acid production showed different trends in the three kinds medium of glucose-rich phase, glucose-rich phase with yeast extract addition, and the fresh medium (Yan et al. 2016). The bacteriostatic effect is obvious in the glucose-rich phase-based medium. However, lactic acid yield in the medium of glucose-rich phase with yeast extract addition is similar as it in the fresh medium. As a result, a final lactic acid titer of 130 g/L with a yield of 0.91 g/g was obtained. Due to the toxicity of [Bmim]BF_4_ to microorganisms, the concentration of [Bmim]BF_4_ that remained in the bottom phase was critical for utilizing the glucose-rich phase. In general, the toxicity of ILs appears to be directly proportional to the length of the alkyl chain and the number of alkyl groups substituted on the cation. ILs with alkyl groups containing 10 and 14 carbon atoms are toxic to bacteria and fungi (Yu et al. 2016). In addition, enzymes are typically inactive in ILs containing the anions NO_3_^−^, CH_3_CO_2_^−^, CF_3_CO_2_^−^, and CF_3_SO_3_^−^, but active in ILs containing the anions BF_4_^−^, PF_6_^−^, and Tf_2_N^−^ (Kaar et al. 2003). Thus, designing ILs with alkyl groups shorter than 10 carbon atoms and BF_4_^−^ anions may help avoid microbial toxicity (Pernak et al. 2003). [Bmim]BF_4_ was toxic to the growth of *Escherichia coli*, *Pichia pastoris*, and *Bacillus* at 1% (v/v), which equals approximately 12.15 g/L (Ganske and Bornscheuer 2006). In this study, only 2% (w/w) [Bmim]BF_4_ remained in the bottom phase after three-stage sugaring-out extraction. The final concentration of [Bmim]BF_4_ and glucose in the bottom phase was 17.70 g/L and 577.96 g/L, respectively. To ferment, the high glucose concentration in the bottom phase must be diluted 5–6 times, with a concentration of [Bmim]BF_4_ less than 3.54 g/L. When the glucose-rich phase was recycled to ferment, the toxicity of residual [Bmim]BF_4_ to cell growth was thus negligible. Furthermore, IL-tolerant microorganisms and microbial communities recently have recently been discovered in environmental samples, and studies have begun to elucidate mechanisms of ILs tolerance (Yu et al. 2016).

Cells, soluble proteins, and pigments were also extracted using a multi-stage sugaring-out extraction. The total removal ratio of cells, soluble proteins, and pigments decreased with increasing extraction stage due to them being distributed to the top phase, as compared to each stage of sugaring-out extraction. As a result, the total removal ratio of cells and pigments from the unfiltered broth was 68.4% and 65.4%, respectively. The filtered broth had a slightly higher removal ratio of cells of 70.6% and a slightly lower removal ratio of pigments of 57.0%. Because of the lack of salting-out and solvent precipitation, the IL-based sugaring-out extraction system performed poorly for soluble protein removal when compared to solvent-based salting-out and sugaring-out extraction. Only about 10% soluble proteins were removed in the three-stage IL-based sugaring-out extraction. The salting-out extraction system of acetone and ammonium sulfate can remove 90.8% soluble proteins from succinic acid fermentation broth (Sun et al. 2014). Furthermore, 61.3% soluble proteins was removed from lactic acid fermentation broth using isopropanol/glucose sugaring-out extraction system (Yan et al. 2016).

Recently, IL-based ATPS have been successfully used in the extraction, concentration, and purification of the most diverse biomolecules, including proteins, enzymes, antioxidants, synthetic and fermentative produced drugs, and biochemicals. However, most ILs are still expensive in comparison with more conventional solvents. In industrial applications, ILs are inevitably mixed with other solvents or products. Therefore, the development of efficient separation and recycling routes is a crucial attempt to decrease costs and their environmental footprint. Some remarkable achievement for ILs recycling was obtained, which supports establishing an IL-based ATPS as a sound basis of greener cost-effective strategies with sustainable development (Claudio et al. 2014). In this study, multi-stage sugaring-out extraction was applied to obtain higher lactic acid extractability. Objectively speaking, multi-stage sugaring-out extraction is more accessible to achieve on a laboratory scale than continuous counter-current sugaring-out extraction. However, in industrial application, continuous counter-current sugaring-out extraction is a feasible and effective strategy for the separation of target products.

## Conclusions

In this study, ionic liquid-based sugaring-out extraction was developed and investigated to separate lactic acid from synthetic solution and lignocellulose-derived fermentation broth. Except for [E_OH_mim]BF_4_, the ILs with BF_4_^−^ and OTF^−^ anion can form aqueous two-phase systems with the aid of saccharides. Due to the decrease in ILs’ kosmotropicity, the ATPS formation ability of ILs could be promoted by increasing the side-chain length of ILs in the order of [Hmim]BF_4_ ≈ [Bmim]BF_4_ ˃ [Emim]BF_4_ with the same kind of saccharides. On the other hand, for the same type of ILs, an ATPS was formed more easily with glucose than with xylose. About 51.8% LA and 92.3% [Bmim]BF_4_ were partitioned to the top phase, and 97.0% glucose was extracted to the bottom phase at a sugaring-out extraction system consisting of 25% (w/w) glucose and 45% (w/w) IL at pH 2.0. The total recovery of LA would increase to 89.0% in three-stage sugaring-out extraction from synthetic solution. In three-stage sugaring-out extraction from filtered and unfiltered fermentation broth obtained via SSCF of acid-pretreated CS, the total recovery of LA was 89.5% and 89.4%, respectively. Meanwhile, the total removal ratio of cells and pigments from the unfiltered broth was 68.4% and 65.4%, respectively. Although the lactic acid extractability for one stage of IL-based sugaring-out extraction is lower than that of organic solvent-based sugaring-out extraction, the glucose-rich phase with low toxicity of ILs coupled with upstream fermentation technology deserves a promising strategy for lactic acid production.

## Data Availability

All data generated or analyzed during this study are included in this article.

## Abbreviations

LA: Lactic acid
ATP: Aqueous two-phase
ATPS: Aqueous two-phase system
IL: Ionic liquid
CS: Corn stover
SSCF: Simultaneous saccharification and co-fermentation

## References

[CR1] Ahmad A, Banat F, Taher H (2020). A review on the lactic acid fermentation from low-cost renewable materials: recent developments and challenges. Environ Technol Innovation.

[CR2] Aydogan O, Bayraktar E, Mehmetoglu U (2011). Aqueous two-phase extraction of lactic acid: optimization by response surface methodology. Sep Sci Technol.

[CR3] Chen YH, Wang YG, Cheng QY, Liu XL, Zhang SJ (2009). Carbohydrates-tailored phase tunable systems composed of ionic liquids and water. J Chem Thermodyn.

[CR4] Choi JH, Kim SH, Moon SH (2002). Recovery of lactic acid from sodium lactate by ion substitution using ion-exchange membrane. Sep Purif Technol.

[CR5] Claudio AFM, Marques CFC, Boal-Palheiros I, Freire MG, Coutinho JAP (2014). Development of back-extraction and recyclability routes for ionic-liquid-based aqueous two-phase systems. Green Chem.

[CR6] Dai JY, Liu CJ, Xiu ZL (2015). Sugaring-out extraction of 2,3-butanediol from fermentation broths. Process Biochem.

[CR7] Dai JY, Ma LH, Wang ZF, Guan WT, Xiu ZL (2017). Sugaring-out extraction of acetoin from fermentation broth by coupling with fermentation. Bioprocess Biosyst Eng.

[CR8] Dai JY, Sun YQ, Xiu ZL (2021). Ionic liquid-based salting-out extraction of bio-chemicals. Chin J Chem Eng.

[CR9] Fu HX, Sun YQ, Teng H, Zhang DJ, Xiu ZL (2015). Salting-out extraction of carboxylic acids. Sep Purif Technol.

[CR10] Ganske F, Bornscheuer UT (2006). Growth of *Escherichia coli*, *Pichia pastoris* and *Bacillus cereus* in the presence of the ionic liquids [BMIM][BF_4_] and [BMIM][PF_6_] and organic solvents. Biotechnol Lett.

[CR11] Han X, Armstrong DW (2007). Ionic liquids in separations. Acc Chem Res.

[CR12] Isikgor FH, Becer CR (2015). Lignocellulosic biomass: a sustainable platform for the production of bio-based chemicals and polymers. Polym Chem.

[CR13] Kaar JL, Jesionowski AM, Berberich JA, Moulton R, Russell AJ (2003). Impact of ionic liquid physical properties on lipase activity and stability. J Am Chem Soc.

[CR14] Komesu A, Maciel MRW, de Oliveira JAR, Martins LHD, Maciel R (2017). Purification of lactic acid produced by fermentation: focus on non-traditional distillation processes. Sep Purif Rev.

[CR15] Lan K, Xu S, Li J, Hu C (2019). Recovery of lactic acid from corn stover hemicellulose-derived liquor. ACS Omega.

[CR16] Li CX, Han J, Wang Y, Yan YS, Xu XH, Pan JM (2009). Extraction and mechanism investigation of trace roxithromycin in real water samples by use of ionic liquid-salt aqueous two-phase system. Anal Chim Acta.

[CR17] Lightfoot EN, Moscariello JS (2004). Bioseparations. Biotechnol Bioeng.

[CR18] Matsumoto M, Takahashi T, Fukushima K (2003). Synergistic extraction of lactic acid with alkylamine and tri-n-butylphosphate: effects of amines, diluents and temperature. Sep Purif Technol.

[CR19] Matsumoto M, Takemori S, Tahara Y (2020). Lactic acid permeation through deep eutectic solvents-based polymer inclusion membranes. Membranes.

[CR20] Meng KX, Zhang GY, Ding CQ, Zhang TY, Yan H, Zhang DP, Fang TQ, Liu MY, You ZC, Yang CH, Shen J, Jin X (2020). Recent advances on purification of lactic acid. Chem Rec.

[CR21] Muller A, Schulz R, Wittmann J, Kaplanow I, Gorak A (2013). Investigation of a phosphate/1-butyl-3-methylimidazolium trifluoromethanesulfonate/water system for the extraction of 1,3-propanediol from fermentation broth. RSC Adv.

[CR22] Oliveira FS, Araujo JMM, Ferreira R, Rebelo LPN, Marrucho IM (2012). Extraction of L-lactic, L-malic, and succinic acids using phosphonium-based ionic liquids. Sep Purif Technol.

[CR23] Pernak J, Sobaszkiewicz K, Mirska I (2003). Anti-microbial activities of ionic liquids. Green Chem.

[CR24] Pratiwi AI, Yokouchi T, Matsumoto M, Kondo K (2015). Extraction of succinic acid by aqueous two-phase system using alcohols/salts and ionic liquids/salts. Sep Purif Technol.

[CR25] Song ZY, Sun YQ, Wei BC, Xiu ZL (2013). Two-step salting-out extraction of 1,3-propanediol and lactic acid from the fermentation broth of *Klebsiella pneumoniae* on biodiesel-derived crude glycerol. Eng Life Sci.

[CR26] Sui H, Zhou JJ, Ma GQ, Niu YQ, Cheng J, He L, Li XG (2018). Removal of ionic liquids from oil sands processing solution by ion-exchange resin. Appl Sci.

[CR27] Sun YQ, Yan L, Fu HX, Xiu ZL (2014). Salting-out extraction and crystallization of succinic acid from fermentation broths. Process Biochem.

[CR28] Sun YQ, Zhang SS, Zhang XX, Zheng YF, Xiu ZL (2018). Ionic liquid-based sugaring-out and salting-out extraction of succinic acid. Sep Purif Technol.

[CR29] Sun YQ, Zhang XX, Zheng YF, Yan L, Xiu ZL (2019). Sugaring-out extraction combining crystallization for recovery of succinic acid. Sep Purif Technol.

[CR30] Sun YQ, Li XY, Wei CX, Qi WB, Xiu ZL (2021). An aptly industrialized bioprocess for lactic acid production from corn stover using thermotolerant microbial consortia. Bioprocess Biosyst Eng.

[CR31] Tang B, Tian M, Row KH (2013). Adsorption of lactic acid onto three ionic liquid-modified porous polymers. J Appl Polym Sci.

[CR32] Trindade JR, Visak ZP, Blesic M, Marrucho IM, Coutinho JAP, Canongia Lopes JN, Rebelo PN (2007). Salting-out effects in aqueous ionic liquid solutions: cloud-point temperature shifts. J Phys Chem B.

[CR33] Wakisaka A, Ohki T (2005). Phase separation of water-alcohol binary mixtures induced by the microheterogeneity. Faraday Discuss.

[CR34] Wang B, Ezejias T, Feng H, Blaschek H (2008). Sugaring-out: a novel phase separation and extraction system. Chem Eng Sci.

[CR35] Wang B, Feng H, Ezeji T, Blaschek H (2008). Sugaring-out separation of acetonitrile from its aqueous solution. Chem Eng Technol.

[CR36] Wang XL, Nie Y, Zhang XP, Zhang SJ, Li JW (2012). Recovery of ionic liquids from dilute aqueous solutions by electrodialysis. Desalination.

[CR37] Wang JF, Luo JQ, Zhang XP, Wan YH (2016). Concentration of ionic liquids by nanofiltration for recycling: filtration behavior and modeling. Sep Purif Technol.

[CR38] Wu B, Zhang YM, Wang HP (2008). Aqueous biphasic systems of hydrophilic ionic liquids plus sucrose for separation. J Chem Eng Data.

[CR39] Wu B, Zhang YM, Wang HP (2008). Phase behavior for ternary systems composed of ionic liquid plus saccharides plus water. J Phys Chem B.

[CR40] Xu SG, Lan KQ, Li JM, He T, Hu CW (2018). Separation of lactic acid from synthetic solutions and the mixture directly derived from corn stover by aqueous two phase extraction. Sep Purif Technol.

[CR41] Yan L, Sun YQ, Xiu ZL (2016). Sugaring-out extraction coupled with fermentation of lactic acid. Sep Purif Technol.

[CR42] Yan L, Sun YQ, Wang XD, Fu HX, Mu Y, Xiu ZL (2018). Partition behavior of monocarboxylic acids in salting-out extraction systems of monohydric alcohols and dipotassium phosphate. Sep Purif Technol.

[CR43] Yankov DS, Trusler JPM, Yordanov BY, Stateva RP (2008). Influence of lactic acid on the formation of aqueous two-phase systems containing poly(ethylene glycol) and phosphates. J Chem Eng Data.

[CR44] Yu CW, Simmons BA, Singer SW, Thelen MP, VanderGheynst JS (2016). Ionic liquid-tolerant microorganisms and microbial communities for lignocellulose conversion to bioproducts. Appl Microbiol Biotechnol.

[CR45] Yuan SF, Hsu TC, Wang CA, Jang MF, Kuo YC, Alper HS, Guo GL, Hwang WS (2018). Production of optically pure l(+)-lactic acid from waste plywood chips using an isolated thermotolerant *Enterococcus faecalis SI* at a pilot scale. J Ind Microbiol Biotechnol.

[CR46] Zhang YQ, Zhang SJ, Chen YH, Zhang JM (2007). Aqueous biphasic systems composed of ionic liquid and fructose. Fluid Phase Equilib.

[CR47] Zhang Y, Qian Z, Liu P, Liu L, Zheng Z, Ouyang J (2018). Efficient in situ separation and production of L-lactic acid by *Bacillus coagulans* using weak basic anion-exchange resin. Bioprocess Biosyst Eng.

